# Transformation products elucidation of forchlorfenuron in postharvest kiwifruit by time-of-flight mass spectrometry

**DOI:** 10.1371/journal.pone.0184021

**Published:** 2017-09-06

**Authors:** Zhiwei Zhang, Zhenhong Gao, Yuan Wang, Yahong Yuan, Jing Dong, Tianli Yue

**Affiliations:** 1 College of Food Science and Engineering, Northwest A&F University, Shananxi, China; 2 College of Food Science and Engineering, Qingdao Agricultural University, Qingdao, China; 3 Laboratory of Quality & Safety Risk Assessment for Agro-products (YangLing), Ministry of Agriculture, Yangling, Shaanxi,China; 4 National Engineering Research Center of Agriculture Integration Test (Yangling), Yangling, Shaanxi,China; 5 Shimadzu International Trading, Beijing, China; Indian Institute of Chemical Technology, INDIA

## Abstract

Forchlorfenuron (1-(2-chloro-4-pyridyl)-3-phenylurea, FCF) is a plant growth regulator, being extensively used for increasing kiwifruit size. The toxicological properties of its may persist in their transformation products (TPs) or even higher toxicity than FCF. TPs elucidation of FCF in postharvest kiwifruit (*Actinidia chinensis*, Chinese gooseberry) by the liquid chromatography ionization hybrid ion trap and time-of-flight mass spectrometry (LC-ESI-IT-TOF/MS) in positive mode was the objective of the present study. Fifteen days after full bloom, kiwifruits were dipped for 5s with high dosage FCF solution (60 mg/L), so that sufficient peaks could be detected. The chemical structure of unknown TPs was analyzed in combination of functions of LCMS-IT-TOF, such as high-accurate MS^n^, formula predictor, metabolite structural analysis software MetID Solution, profiling solution metabolomics software, and neutral loss, characteristic isotopic patterns of chlorine, the fragmentation pattern and retention time of standard substances, nitrogen rule, chemical components of kiwifruit. Total 17 TPs were detected via comparisons of their accurate MS^n^ data of commercial analytical standards and synthesized standards with high purity, such as 4-amino-2-chloropyridine, phenylurea, 2-hydroxy-FCF, 1-(2-chloro-6-((3, 4, 5-trihydroxy-6-(hydroxymethyl) tetrahydro-2H-pyran-2-yl) oxy) pyridin-4-yl)-3-phenylurea, 1, 3-bis (2-chloropyridin-4-yl) urea, 1,3-diphenylurea, 1-(2-chloropyridin-4-yl)urea, FCF-2-O-β-D-glucoside, and so on. The major transformation pathways of FCF in kiwifruit were biochemical and photochemical cleavage pathway. The experimental results indicate that LCMS-IT-TOF is powerful and effective tool for identification of FCF TPs.

## Introduction

FCF is a plant growth regulator, being extensively used for increasing fruit size and weight in watermelon, kiwifruit, grape and apple[1–4]. In China, the incidence of FCF had caused an adverse impact on the kiwifruit industry. Residue analysis and dissipation of FCF in fruit and vegetable had already been studied by high-performance liquid chromatography with ultraviolet detection (HPLC/UV)[5], liquid chromatography-tandem mass spectrometry[6–8], liquid chromatography time-of-flight mass spectrometry (LC/TOF-MS)[9]. An enzyme-linked immunosorbent assay (ELISA) had been developed for the determination of FCF in fruit[10–12]. Evaluation of the new activity of FCF in the product was reported by Australian pesticides and veterinary medicines authority. FCF has low oral toxicity in rats with an LD50 of 4940 mg/kg bw in male rats and 4899 mg/kg bw in females[13]. In recent years, some researches indicated that TPs of agrochemistry could be more toxic than the parent molecule[14,15]. So FCF and its TPs could be a potential health hazard. However, the detection of TPs was a difficult work to do, due to the lack of standards[16]. Currently, the time-of-flight (TOF) mass analyzers had been used to detect TPs of agrochemistry[17–21].

LCMS-IT-TOF (Shimadzu) is a type of mass spectrometer that combines ion trap and TOF (time-of-flight) technologies. The instrument possesses some advantages and function, such as high accuracy MS^n^, Formula Predictions software, MetID solution software[22]. LCMS-IT-TOF/MS had been successfully applied to the identification of metabolites of FR429[23], herbal homologs, strictosamide, phencynonate[24,25],. In this paper, LCMS-IT-TOF had been employed to identify the major TPs of FCF in postharvest kiwifruit.

## Material and methods

### Ethics statement

I state clearly that no specific permissions were required for these locations/activities, because this site is a normal kiwifruit orchard without protected wildlife and protected area of land or sea. The authors confirm that the field studies did not involve endangered or protected species. The study was approved by Shaanxi Bairui Kiwi Fruit Research Institute Co. Ltd.

FCF (purity>99%, M0), 4-amino-2-chloropyridine (97%, ST1), phenylurea (97%, ST2) were provided by Sigma-Aldrich. FCF-4-O-β-D-glucoside (>95%, ST3), FCF-3- O-β-D -glucoside (>95%, ST4), 4-hydroxy-FCF (>95%, ST5) and 3-hydroxy-FCF (>95%, ST6) come from chemical synthesis [28]. High performance liquid chromatography (HPLC) grade methanol and acetonitrile were provided by Sigma-Aldrich. Formic acid and ethyl acetate were of analytical grade. HPLC grade water was obtained in a Milli-Q-Plus ultrapure water system from Millipore.

### Plant material

The trial was carried out in Xi'an city, Shaanxi province, China, in 2013~2015, in a 7-year-old kiwifruit orchard of Hayward from Shaanxi Bairui Kiwi Fruit Research Institute Co. Ltd (34°03′N, 108°25′E). The vines were spaced 5×4 m, trained to the T-bar trellis system. Twenty vines were selected, uniform in vegetative and reproductive characteristics. Fifteen days after full bloom, all fruitlets of fifteen vines were dipped for 5 s with FCF (ethanol solvent, 60 mg/L), sufficient peaks could be detected in high dosage, while the fruit of the other five vines were dipped with water only (blank). Storage time of postharvest kiwifruit is one month.

### Sample preparation

Extraction of FCF and TPs from kiwifruit samples was carried out according to the QuEChERS method[26,27]: (1) FCF treated samples were placed in a blender and chopped. (2) 300 g of thoroughly homogenized sample was placed into a 1000 mL glass beaker, mixed thoroughly with 300 mL of acetonitrile and 120 g anhydrous magnesium sulfate, and 30 g anhydrous sodium acetate using ultrasonic-assisted extraction for 40 min. The homogenate was allowed to settle and the supernatant was filtered through a filter paper into a 1000 mL rotary-evaporation flask. The solid residue was washed twice with 60 mL of acetonitrile. A rotary evaporator set at 50°C and 250 mbar was used to evaporate the extract to less than 5 mL, and then, the extract was passed to a graduated conical tube (15 mL) and evaporated to dryness at 50°C. The sample was reconstituted in water: acetonitrile (1:1) and filtered through a 0.22 *μ*m filter[28].

### Chromatographic conditions

LC analysis was conducted on a LC-20AD system from Shimadzu (Kyoto, Japan). Chromatographic separation was achieved on a Shim-pack XR ODS column (2.2*μ*m 3.0 mm×75 mm from Shimadzu). The mobile phase (0.2 mL min^-1^) consisted of solvent A (0.1% formic acid in acetonitrile) and B (0.1% formic acid in water). Elution condition was performed with a linear gradient 95~30% B from 0 to 5 min, 30~0% B from 5 to10 min, retained until 1 min then quickly returned to initial 95% B and maintained for 20 min for column balance [28].

MS^n^ analyses were conducted on a Shimadzu LC-IT-TOF/MS (Shimadzu, Kyoto, Japan) equipped with an electrospray ionization (ESI) source operated in positive mode, and the optimized operating conditions were as follows: detector voltage, 1.6 kV; curved desolvation line (CDL) temperature, 200°C; heat block temperature, 200°C; nebulizing gas flow, 1.5 L/min; drying gas (N_2_) pressure, 100kPa; scan range, *m/z* 100–1000 for MS^1^, *m/z* 100–800 for MS^2^, *m/z* 100–800 for MS^3 [^^28^^]^.

The identification process of FCF and its TPs involves four procedural steps: (1) sample preparation (2) elucidated the fragment pattern of FCF and its 6 TPs standard substance. (3)The chemical structure of unknown TPs was analyzed in combination of functions of LCMS-IT-TOF, such as high-accurate MS^n^, formula predictor, metabolite structural analysis software MetID Solution, profiling solution metabolomics software, and neutral loss, characteristic isotopic patterns of chlorine, the fragmentation pattern and retention time of standard substances, nitrogen rule, chemical components of kiwifruit. Some suspected TPs peaks which had the same MS^n^ and fragment pattern with FCF and its 6 TPs standard substance were found by full scan analysis in positive mode. (4) concluded their chemistry structure.

## Results and discussion

### Fragmentation mass spectra for standard substances of (possible) transformation products

**Fig 1:**
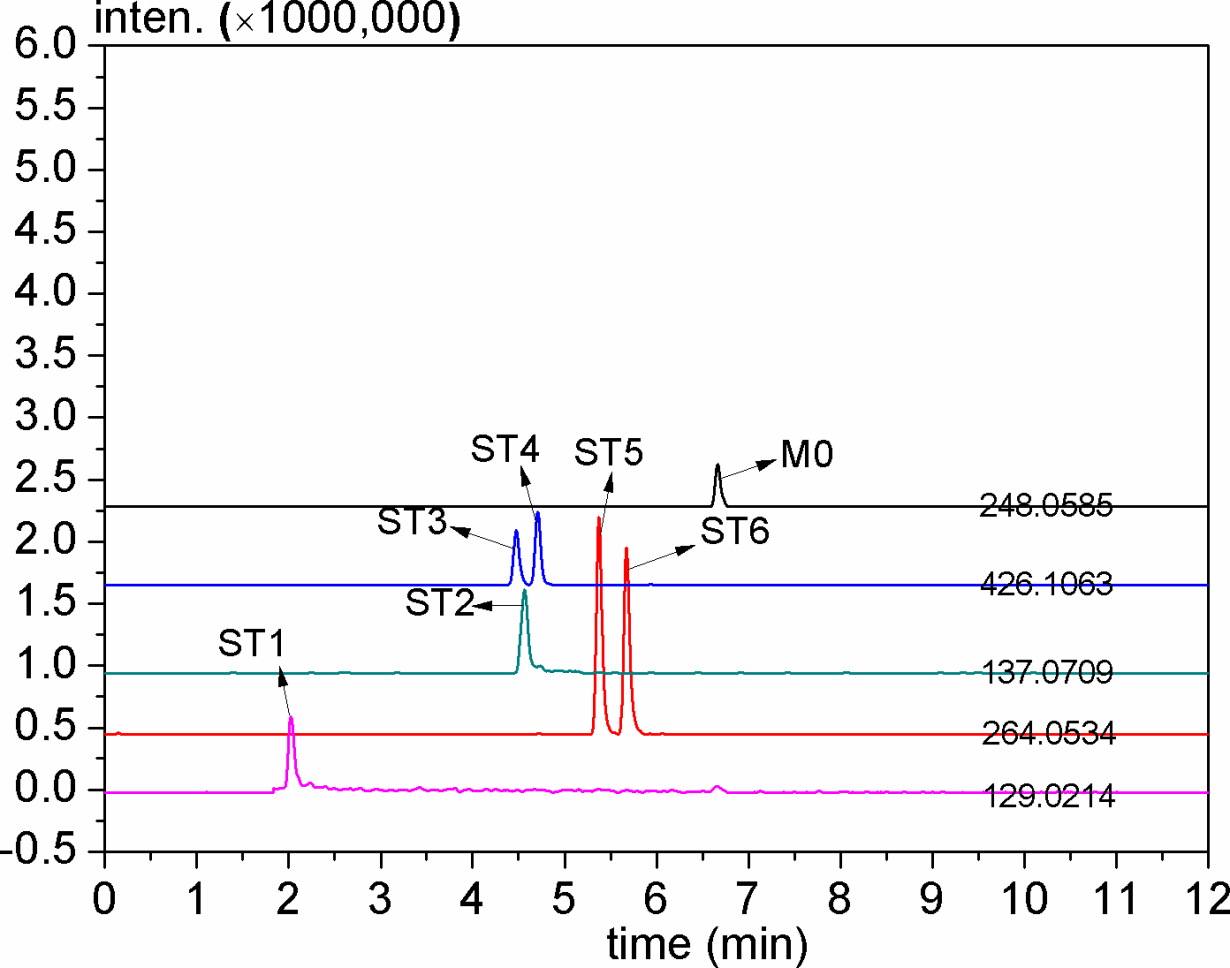
Extract ion chromatograms of 6 TPs standard substance and M0.

**Fig 2:**
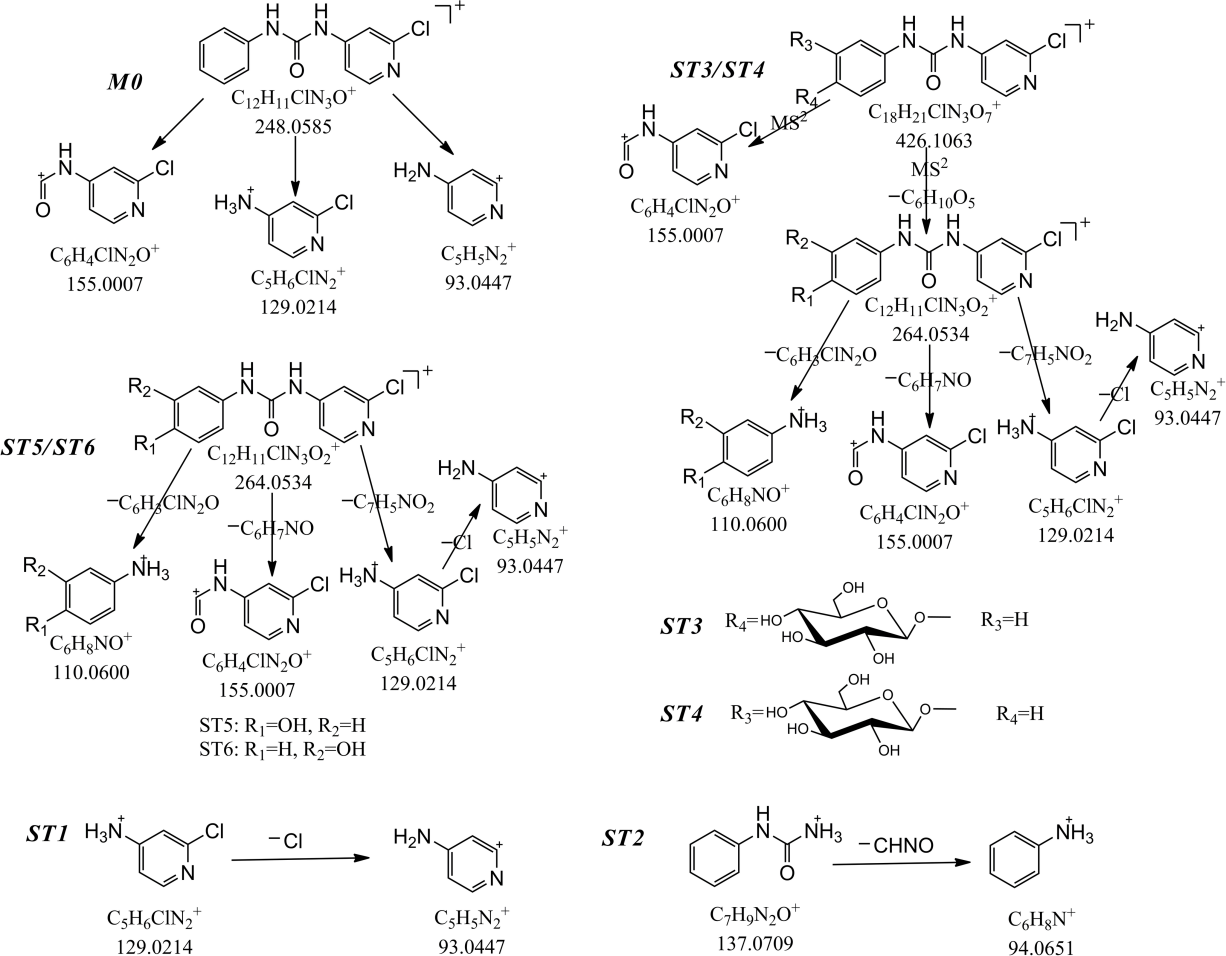
The proposed fragmentation pattern of M0 and 6 TPs standard substance.

Forchlorfenuron (M0) and 6 TPs (10 μg mL^-1^) standard substance prepared respectively in methanol was used for the fragmentation pattern study in positive ion mode. Fig 1 shows extract ion chromatograms of 6 TPs standard substance and M0. Table 1 shows analytical information of the 6 TPs standard substance, including retention time, elemental compositions, and mass error in positive ion mode. Fig 2 shows the proposed fragmentation pattern of M0 and 6 TPs standard substance [28].

**Table 1:** The MS^n^ data of the 6 TPs and M0 in positive ion mode.

ST	RT (min)	Theoretical Value [M+H]^+^	Elemental compositions	MS^n^	MS^n^ [M+H]^+^	Error (ppm)
ST1	2.003	129.0214	C_5_H_5_ClN_2_	MS^1^	***129*.*0207***	-5.4
				MS^2^	93.0492	
ST2	4.560	137.0709	C_7_H_8_N_2_O	MS^1^	***137*.*0705***	-2.9
				MS^2^	94.0688	
ST3	4.470	426.1063	C_18_H_20_ClN_3_O_7_	MS^1^	***426*.*1045***	-4.2
				MS^2^	***264*.*0514***, 155.0004,129.0162	
				MS^3^	154.9999, 129.0218, 110.0563, 93.0441	
ST4	4.710	426.1063	C_18_H_20_ClN_3_O_7_	MS^1^	***426*.*1035***	-6.6
				MS^2^	***264*.*0515***, 154.9991, 129.0213	
				MS^3^	155.0008, 129.0212, 110.0563, 93.0436	
ST5	5.370	264.0534	C_12_H_10_ClN_3_O_2_	MS^1^	***264*.*0514***	-7.6
				MS^2^	154.9992, 129.0203, 110.0575, 93.0419	
ST6	5.670	264.0534	C_12_H_10_ClN_3_O_2_	MS^1^	***264*.*0515***	-7.2
				MS^2^	154.9986, 129.0215, 110.0605, 93.0429	
M0	6.660	248.0585	C_12_H_10_ClN_3_O	MS^1^	***248*.*0572***	-5.2
				MS^2^	155.0001, 129.0208, 93.0436	

Standard Substance = ST, retention time = RT

### Identification of forchlorfenuron transformation products in postharvest kiwifruit

**Fig 3:**
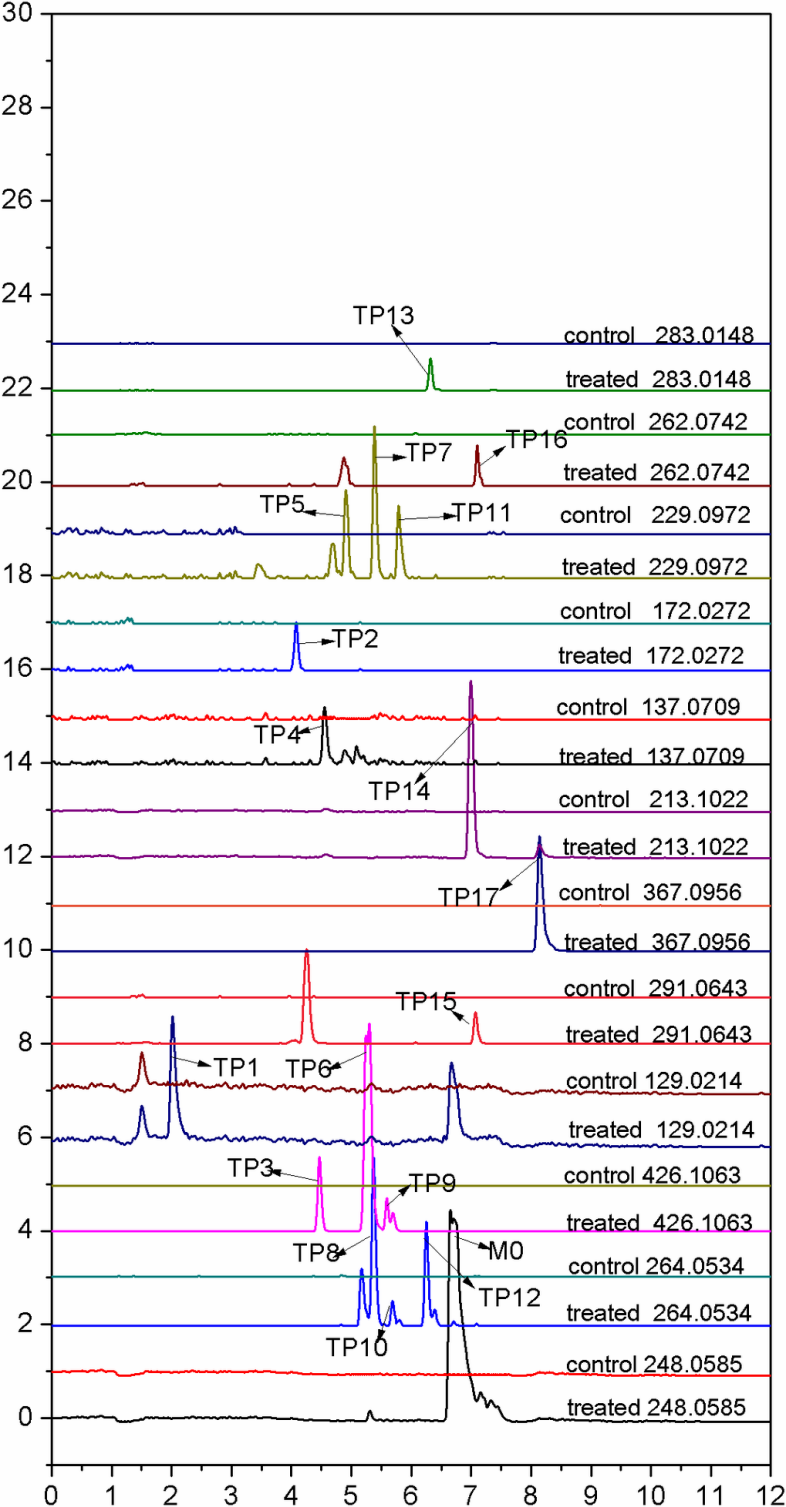
Extract ion chromatograms of 17 TPs.

**Fig 4:**
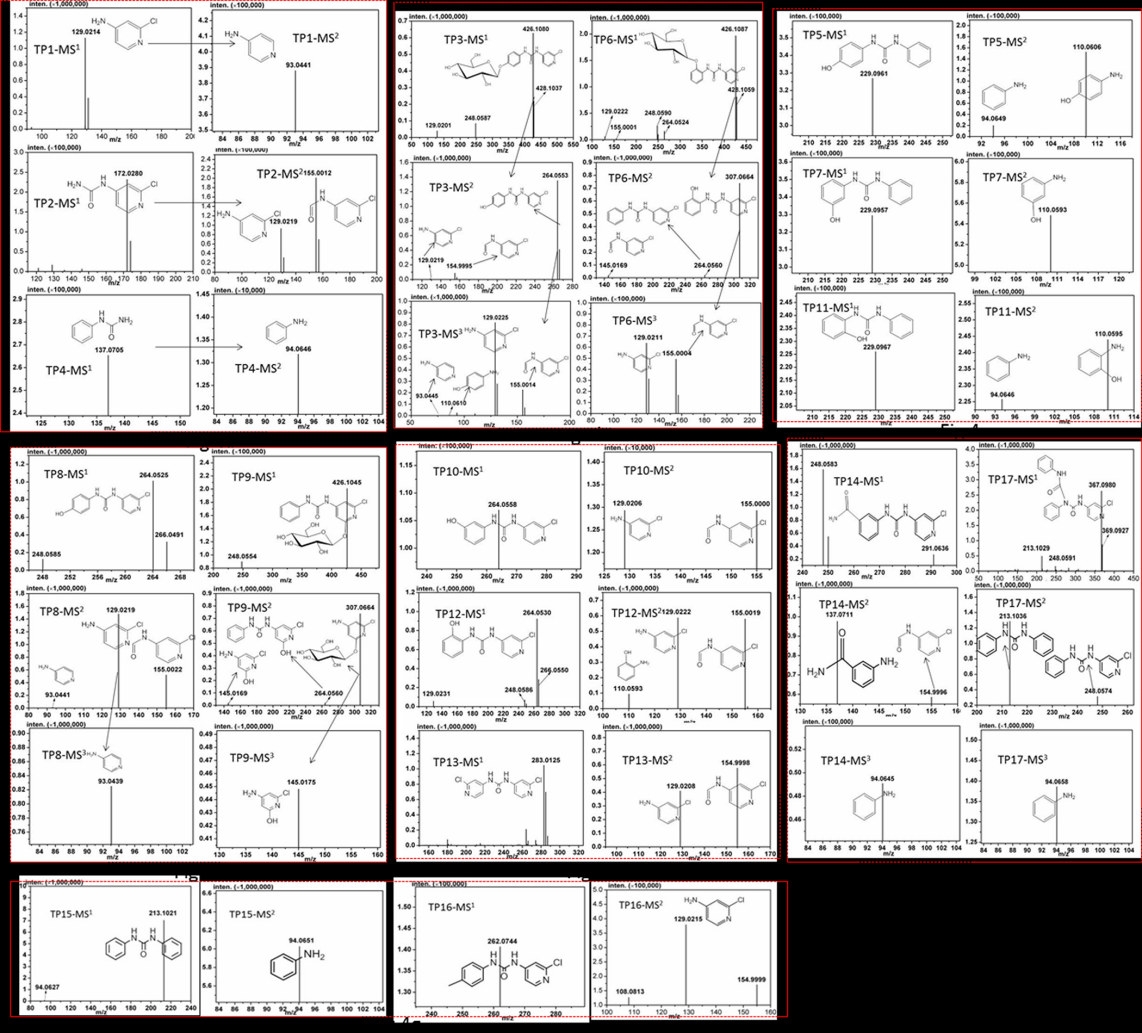
MS^n^ on FCF TPs and their proposed structures in positive ion mode.

One month after kiwifruit posthavest, 17 TPs were deduced by analysis of LCMS-IT-TOF. Fig 3 shows extract ion chromatograms of 17 TPs. Table 2 displays the data of the MS^n^ data of 17 TPs. Fig 4A–4G show TOF-MS/MS spectrum on TPs and their proposed structures in positive ion mode.

**Table 2:** The MS^n^ data of the 17 TPs.

TPs	RT (min)	Elemental compositions	MS^n^	MS^n^ [M+H]^+^	Error (ppm)
TP1	2.001	C_5_H_5_ClN_2_	MS^1^	129.0214	0
			MS^2^	93.0441	-6.5
TP2	4.085	C_6_H_6_ClN_3_O	MS^1^	172.0280	4.7
			MS^2^	155.0012, 129.0219	3.2, 3.9
TP3	4.467	C_18_H_20_ClN_3_O_7_	MS^1^	426.1080	4.0
			MS^2^	***264*.*0553***, 154.9995, 129.0219	7.2, -7.7, 3.9
			MS^3^	155.0014, 129.0225, 110.0610, 93.0445	4.5, 8.5, 9.1, -2.2
TP4	4.563	C_7_H_8_N_2_O	MS^1^	137.0705	-2.9
			MS^2^	94.0646	-5.3
TP5	4.900	C_13_H_12_N_2_O_2_	MS^1^	229.0961	-4.8
			MS^2^	110.0606, 94.0649	5.5, -2.1
TP6	5.355	C_18_H_20_ClN_3_O_7_	MS^1^	426.1087	5.6
			MS^2^	***264*.*0539***, 248.0591, 155.0000	1.9, 2.4, -4.5
			MS^3^	155.0004, 129.0211	-1.9, -2.3
TP7	5.363	C_13_H_12_N_2_O_2_	MS^1^	229.0957	-6.5
			MS^2^	110.0593	-6.4
TP8	5.370	C_12_H_10_ClN_3_O_2_	MS^1^	264.0525	-3.4
			MS^2^	155.0022, ***129*.*0219***, 93.0441	9.7, -3.9, -6.5
			MS^3^	93.0439	-8.6
TP9	5.610	C_18_H_20_ClN_3_O_7_	MS^1^	426.1045	-4.2
			MS^2^	***307*.*0664***, 264.0560, 145.0169	-8.8, 9.8, 4.1
			MS^3^	145.0175	8.3
TP10	5.668	C_12_H_10_ClN_3_O_2_	MS^1^	264.0558	9.1
			MS^2^	155.0000, 129.0206	-4.5, -6.2
TP11	5.767	C_13_H_12_N_2_O_2_	MS^1^	229.0967	-2.2
			MS^2^	110.0595, 94.0646	-4.5, -5.3
TP12	6.249	C_12_H_10_ClN_3_O_2_	MS^1^	264.053	-1.5
			MS^2^	155.0019, 129.0222, 110.0593	7.7, 6.2, -6.4
TP13	6.269	C_11_H_9_Cl_2_N_4_O	MS^1^	283.0125	-8.1
			MS^2^	154.9998, 129.0208	-5.8, -4.7
TP14	7.030	C_13_H_12_ClN_4_O_2_	MS^1^	291.0636	-2.4
			MS^2^	154.9996, ***137*.*0711***	-7.1, 1.5
			MS^3^	94.0645	-6.4
TP15	6.987	C_13_H_12_N_2_O	MS^1^	213.1021	-0.5
			MS^2^	94.0651	0
TP16	7.086	C_13_H_12_ClN_3_O	MS^1^	262.0744	0.8
			MS^2^	154.9999, 129.0215	-5.2, 0.8
TP17	8.119	C_19_H_16_ClN_4_O_2_	MS^1^	367.0980	6.5
			MS^2^	248.0574, ***213*.*1036***	-4.4, 6.6
			MS^3^	94.0658	7.4

Retention time = RT

The molecular ion of TP1 showed the predominant protonated molecule ion [M+H]^+^ at *m/z* 129.0214 and was calculated as C_5_H_5_ClN_2_ (error, 0 ppm) by the Formula Predictor software. The difference of retention time between TP1 and ST1 was 0.002 min. So, TP1 was identified as ST1.

TP2 (see Fig 4A, Table 2) gave rise to the protonated molecule at *m/z* 172.0280 with a retention time of 4.085 min, and it was calculated as C_6_H_6_ClN_3_O (error, 4.7 ppm) by the Formula Predictor software. The MS^2^ products ions at *m/z* 155.0012 (error, 3.2 ppm), *m/z* 129.0219 (error, 3.9 ppm) were characteristic fragment ions of M0, and the product ion at *m/z* 155.0012 was formed through neutral losing of NH_3_. Thus, TP2 was identified as 1-(2-chloropyridin-4-yl)urea. The proposed structure and the fragment pathway for TP2 are shown in Fig 4A.

TP3 (see Fig 4B, Table 2, *m/z* 426.1080), eluted at 4.467 min, showed a ^37^Cl signal at *m/z* 428.1037, two fragment ions of the MS^1^ at *m/z* 248.0587, *m/z* 129.0201, and the fragment ion at *m/z* 248.0587 was the characteristic ion of M0. The molecular ion of TP3 gave rise to three main MS^2^ ions at *m/z* 264.0553(error, 7.2 ppm), *m/z* 154.9995(error, -7.7 ppm), *m/z* 129.0219(error, 3.9 ppm), and the fragment ion at *m/z* 264.0553 could lead to four MS^3^ product ions at *m/z* 155.0014(error, 4.5 ppm), *m/z* 129.0225(error, 8.5 ppm), *m/z* 110.0610(error, 9.1 ppm), *m/z* 93.0445(error, -2.2 ppm), also the product ion at *m/z* 264.0553 was calculated as C_12_H_10_ClN_3_O_2_ (error,7.2 ppm) by the Formula Predictor software. Thus, the product ion at *m/z* 426.1068 was concluded as the glycosylation product of M0. TP3 showed the same fragment pathway and retention time as ST3. Therefore, TP3 was identified as ST3.

TP4, eluted at 4.563 min, showed the predominant protonated molecule ion [M+H]^+^ at *m/z* 137.0705, the mass error was -2.9 ppm, and the retention time error was 0.003 min. TP4 yielded a main MS^2^ ions at *m/z* 94.0646 (error, -5.3 ppm). So, TP4 was identified as ST2.

TP5, TP7, TP11 (Fig 4C, Table 2) showed respectively the protonated molecular ion [M+H]^+^ at *m/z* 229.0961(error, -4.8 ppm), *m/z* 229.0957(error, -6.5 ppm), *m/z* 229.0967(error, -2.2 ppm), which were respectively 15.9939 Da (O), 15.9935 Da (O), 15.9945 Da (O) higher than the protonated molecule of TP15 (*m/z* 213.1022). They were calculated as C_13_H_12_N_2_O_2_ by the Formula Predictor software. TP5, TP7, TP11 shared the similar MS^2^ fragment ions at *m/z* 110.0606 (error, 5.5 ppm), *m/z* 110.0593 (error, -6.4 ppm), *m/z* 110.0595 (error, -4.5 ppm). TP5, TP11 shared the similar MS^2^ fragment ions at *m/z* 94.0649 (error, -2.1 ppm), *m/z* 94.0646 (error, -5.3 ppm). Thus, TP5, TP7, TP11 were elucidated by the hydroxylation product of TP14. According to the polar order, we could infer hydroxy position on the benzene ring, TP5, TP7, TP11 is respectively 1-(4-hydroxy-phenyl)–3–phenylurea, 1-(3-hydroxy-phenyl)–3–phenylurea, 1-(2-hydroxy-phenyl)–3–phenylurea. The proposed chemical structure and the fragment pathway for TP5, TP7, TP11 are respectively shown in Fig 4C.

TP6 showed the protonated molecular ion [M+H]^+^ at *m/z* 426.1087 (see Fig 4B, Table 2, error, 5.6 ppm), eluted at 5.355 min, yielded three MS^2^ ions at *m/z* 264.0539(error, 1.84 ppm), *m/z* 248.0591(error, 2.4 ppm), *m/z* 155.0000(error, -4.5 ppm), and the product ion at *m/z* 264.0539 lead to two MS^3^ ions at *m/z* 155.0004(error, -1.9 ppm), *m/z* 129.0211(error, -2.3 ppm). The fragment ions at *m/z* 264.0539, *m/z* 248.0591 were calculated respectively as C_12_H_10_ClN_3_O_2_, C_12_H_10_ClN_3_O by the Formula Predictor software, and the fragment ions at *m/z* 264.0539 had the same daughter ions at *m/z* 155.0004/129.0211 with ST5/ST6, and also the MS^2^ product ion at *m/z* 264.0539 was formed through neutral losing *m/z* 162.0523 (C_6_H_10_O_5_). Thus, we concluded TP6 was the glycosylation product of M0. As can be seen in Fig 4B, the MS^1^ of TP6 yield five fragment ions at *m/z* 264.0524, *m/z* 248.0590, *m/z* 155.0001, *m/z* 129.0222, *m/z* 110.0572, the position of glycosylation for TP6 was concluded in the benzene ring. But the retention time of TP6 had the larger difference with ST3/ST4. Thus, we concluded TP6 was isomers of ST3/ST4, and indentified as FCF-2-O-β-D-glucoside. The proposed structure and the fragment pathway for TP6 are shown in Fig 4B.

TP8, TP10 (see Fig 4D, Fig 4E,Table 2) and TP12 exhibited respectively the protonated molecular ion [M+H]^+^ at *m/z* 264.0525(error, -3.4 ppm), *m/z* 264.0558(error, 9.1 ppm), *m/z* 264.0530(error, -1.5 ppm), which were respectively 15.994 Da, 15.9973 Da, 15.9945 Da (O) higher than the protonated molecule of M0 (*m/z* 248.0585), and they were calculated as C_12_H_10_ClN_3_O_2_ according to the accurate mass by the Formula Predictor software. Thus they could be preliminary concluded as the hydroxylation product of M0. TP8, TP10 and TP12 shared the same MS^2^ fragment ions at *m/z* 155.0007, *m/z* 129.0214, *m/z* 110.0600 with ST5/ST6. The retention time error between TP8 and ST5, TP10 and ST6 were respectively 0min, 0.003min. Therefore, TP8 was identified as ST5, TP10 was identified as ST6. As ST5/ST6 was respectively 4-hydroxy-FCF and 3-hydroxy-FCF, therefore, TP12 was identified as 2-hydroxy-FCF. The chemical structure and the fragment pathway of TP12 had been concluded and shown in Fig 4E.

The protonated molecular ion of TP9 (see Fig 4D, Table 2, *m/z* 426.1045, error -4.2 ppm), retention time 5.610 min, yielded three MS^2^ ions at *m/z* 307.0664(error, -8.8 ppm), *m/z* 264.0560 (error, 9.8 ppm), *m/z* 145.0169 (error, 4.1 ppm). As can be seen in Table 1, the fragment ion at *m/z* 248.0585, *m/z* 264.0540 was respectively the characteristic ions of M0, ST5, ST6, and the fragment ion at *m/z* 264.0564 is 15.9979 Da (O) higher than that of *m/z* 248.0585 (M0), also the MS^2^ ion at *m/z* 264.0564 was formed through neutral losing 162.0510 (C_6_H_10_O_5_). Thus TP9 was the glycosylation product of ST5/ST6. The product ion at *m/z* 307.0664 could lead to the MS^3^ ion at *m/z* 145.0175 by the neutral loss of 162.0489 (C_6_H_10_O_5_), and the fragment ion at *m/z* 145.0175 was 15.9961 Da (O) higher than the fragment ion at *m/z* 129.0214 of ST1. Thus, we conclude the hydroxylated position was located on pyridine ring of M0. Thus, TP9 was identified as 1-(2-chloro-6-((3, 4, 5-trihydroxy-6-(hydroxymethyl) tetrahydro-2H-pyran-2-yl) oxy) pyridin-4-yl)-3-phenylurea.The proposed chemical structure and the fragment pathway for TP9 were shown in Fig 4D.

TP13 (see Fig 4E, Table 2), m/z 283.0125(error, -8.1 ppm) lead to two main MS^2^ ions at m/z [M+H]^+^ *m/z* 154.9998 (error, -5.8 ppm), *m/z* 129.0208 (error, -4.7 ppm), which were the characteristic fragment ions of M0. Meanwhile, we found 3 molecular ions at *m/z* 283.0162, *m/z* 285.0132, *m/z* 287.0113 were present in relative intensities of about 9:6:1 in MS^1^ ions, which was the natural abundance ratio of ^35^Cl and ^37^Cl. Thus, we deduced that there were the two chlorine atoms in TP13. The fragment ion at *m/z* 154.9998 was generated by the neutral loss of *m/z* 128.0127 Da (C_5_H_5_ClN_2_). On the other hand, the fragment ion at *m/z* 129.0208 was generated by the neutral loss of *m/z* 153.9917 Da (C_6_H_3_ClN_2_O). TP13 was calculated as C_11_H_8_Cl_2_N_4_O by the Formula Predictor software. Thus, TP13 was identified as 1, 3-bis (2-chloropyridin-4-yl) urea. The chemical structure and the fragment pathway are shown in Fig 4E.

TP14 (see Fig 4F, Table 2) showed the protonated molecular ion [M+H]^+^ at *m/z* 291.0636, was 43.0051 Da higher than that of the molecular ion of M0 (*m/z* 248.0585) and the ratio of the relative intensities of the principal isotopes, A to A+2 was approximately 3:1, suggesting the presence of a chlorine atom (the natural abundance of ^35^Cl: ^37^Cl is 3:1). The neutral loss of 43.0051 Da was calculated as HNCO by the Formula Predictor software. The MS^2^ (*m/z* 137.0711, error, 1.5 ppm) was formed through neutral losing of *m/z* 153.9932 (C_6_H_3_ClN_2_O). Thus, we concluded the fragment ion at *m/z* 137.0711 was the combination of HNCO and aniline, and its combination position was located on amino of aniline. Further analysis showed the fragment ion at *m/z* 137.0711 was formed by the reaction of aniline and amino acid in kiwifruit sample. Thus, TP14 was identified as 1-carbamoyl-3-(2-chloropyridin-4-yl) -1-phenylurea. The chemical structure and the fragment pathway of TP14 are shown in Fig 4F.

TP15 (see Fig 4G, Table 2, *m/z* 213.1021, error, -0.5 ppm) was calculated as C_13_H_12_N_2_O by the Formula Predictor software according to the accurate mass measurement. As can be seen in Fig 4G, the molecular ion of TP15 showed the major product ions at *m/z* 94.0651 (error, 0 ppm). Thus, TP15 was identified as 1, 3-diphenylurea, and its chemical structures and fragment pattern were shown in Fig 4F.

TP16 (see Fig 4G, Table 2) was detected at the retention time of 7.086 min and gave a protonated molecule ion at *m/z* 262.0744 (error, 0.8 ppm), and there were two characteristic fragment ions at *m/z* 154.9999, *m/z* 129.0215 of M0. The protonated molecule at *m/z* 262.0744 was 14.0157 Da (CH_2_) higher than that of M0, and the product ion of *m/z* 108.0813 was 14.0162 Da (CH_2_) higher than that of phenylamine, and also the MS^2^ ion at *m/z* 108.0813 was generated through the neutral loss of C_6_H_3_ClN_2_O. Thus, we concluded the position of CH_2_ was located at the benzene ring, and it could be 1-(2-chloropyridin-4-yl)-3-(o-tolyl) urea or 1-(2-chloropyridin-4-yl)-3-(m-tolyl) urea or 1-(2-chloropyridin-4-yl)-3-(p-tolyl) urea. Its accurate structure need to be further identified by the reference standards. We tentatively concluded the proposed structure and the fragment pathway of TP16 were shown in Fig 4G.

TP17 (see Fig 4F, Table 2) showed the protonated molecular ion [M+H]^+^ at *m/z* 367.0980 (a ^37^Cl isotope signal of *m/z* 369.0927), and three main MS^2^ ions at *m/z* 248.0574 (error, -4.4 ppm), *m/z* 213.1036 (error, 6.6 ppm) and the fragment ion at *m/z* 213.1036 could lead to one MS^3^ product ions at *m/z* 94.0658. The products ions at *m/z* 248.0574, *m/z* 213.1036 were calculated as respectively C_12_H_10_ClN_3_O (M0), C_13_H_12_N_2_O (TP15) by Formula Predictor software. Thus, TP17 was a reaction product of M0 and TP15 and identified as 3-(2-chloropyridin-4-yl)-1-phenyl-1- (phenylcarbamoyl)urea. The chemical structures of TP17 and its fragment pattern were shown in Fig 4F.

### Degradation and metabolism pathway of forchlorfenuron in kiwifruit

**Fig 5:**
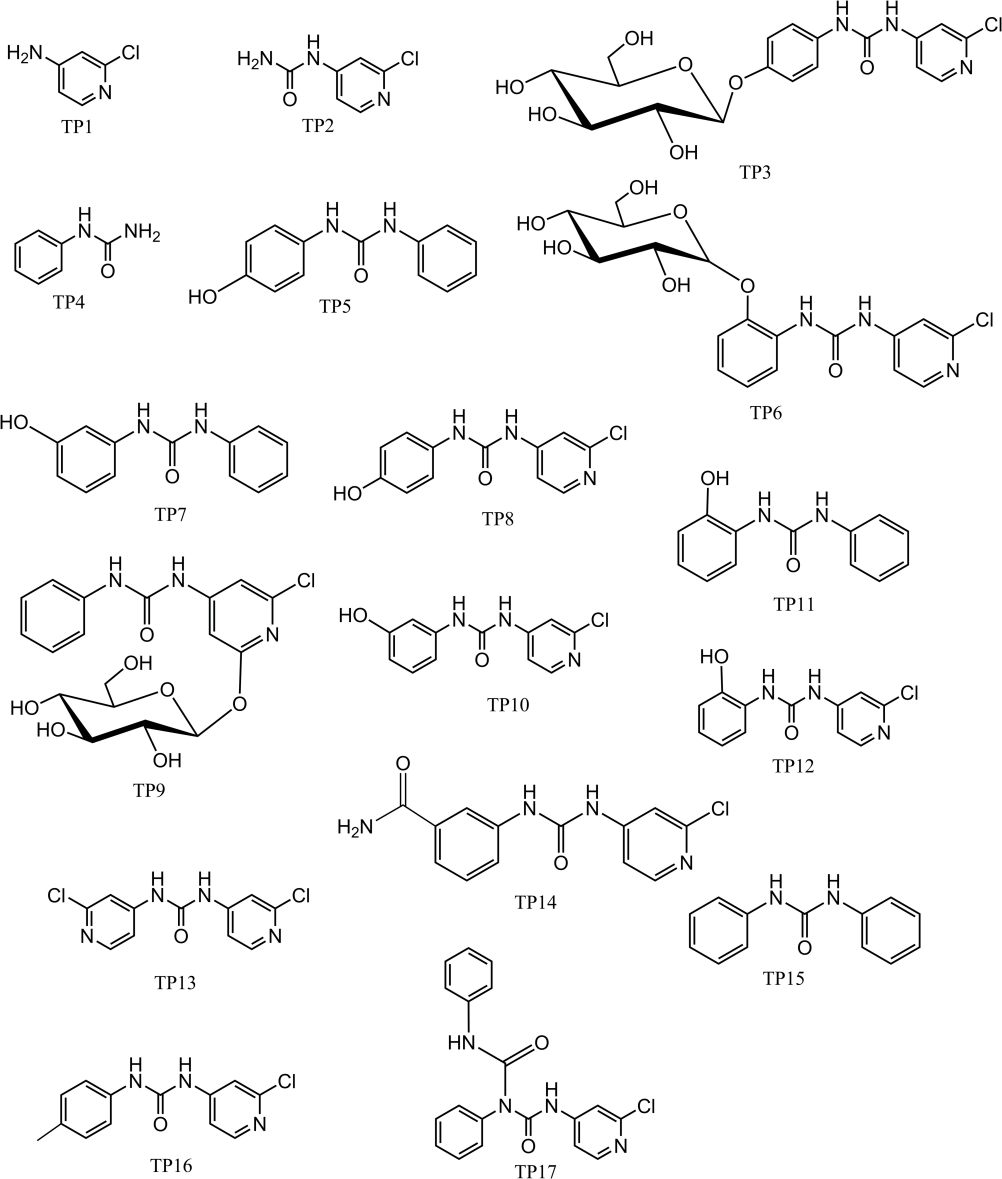
The proposed chemical structure of 17 TPs.

**Fig 6:**
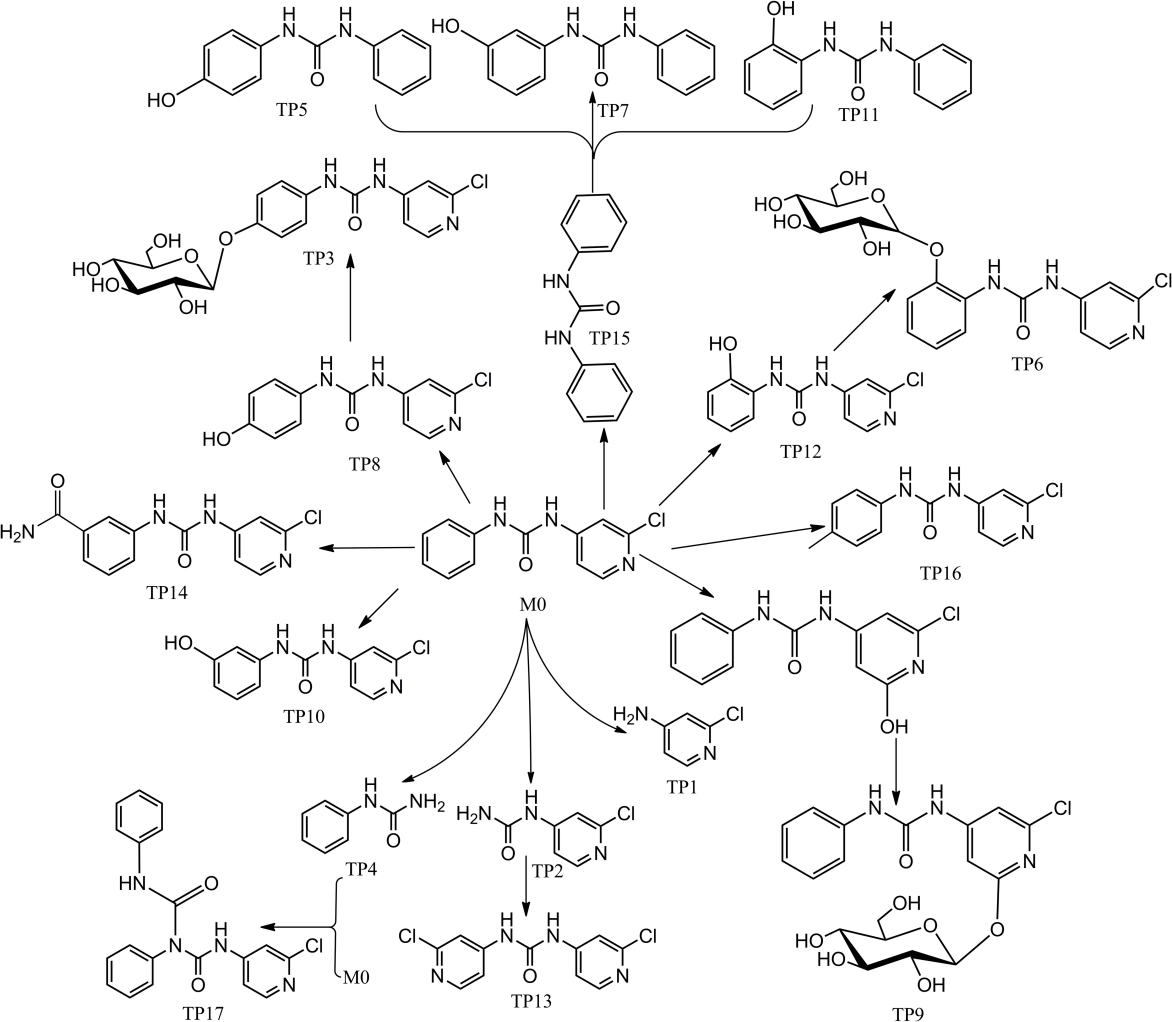
Proposed degradation and metabolism pathway of FCF in kiwifruit.

According to the deduced structure formulas of the 17 TPs (Fig 5), the degradation and metabolism pathway of FCF in kiwifruit were proposed and are shown in Fig 6. The present study showed that 17 TPs could be found and determined, several of which were discovered for the first time. These data indicate some metabolic pathways of FCF, such as, FCF was cleaved to TP1, TP2, TP4, its hydroxylated product is TP8, TP10, TP12, furthermore, its glycosylation product is TP3, TP6, TP9.

## Conclusions

Total 17 TPs of FCF in postharvest kiwifruit were detected by LCMS-IT-TOF, and the main transformation pathways were hydroxylation, glycosylation, methylation, cleavage, oxidation, reduction, and so on. The experimental results indicate that LCMS-IT-TOF is powerful and effective tool for identification of plant growth regulation TPs.
